# Blood Purification in Hepatic Dysfunction after Liver Transplant or Extensive Hepatectomy: Far from the Best-Case Scenarios

**DOI:** 10.3390/jcm13102853

**Published:** 2024-05-12

**Authors:** Rita Gaspari, Paola Aceto, Giorgia Spinazzola, Edoardo Piervincenzi, Maurizio Chioffi, Felice Giuliante, Massimo Antonelli, Alfonso Wolfango Avolio

**Affiliations:** 1 Department of Basic Biotechnological Science, Intensive Care and Peri-Operative Clinics, Università Cattolica del Sacro Cuore, 00168 Rome, Italy; rita.gaspari@unicatt.it (R.G.); massimo.antonelli@unicatt.it (M.A.); 2 Department of Emergency, Anesthesiological and Reanimation Sciences, Fondazione Policlinico Universitario Agostino Gemelli IRCCS, 00168 Rome, Italy; giorgia.spinazzola@policlinicogemelli.it (G.S.); edoardo.piervincenzi@policlinicogemelli.it (E.P.); maurizio.chioffi@gmail.com (M.C.); 3 Department of Gastroenterological, Endocrine, Metabolic and Nephro-Urological Sciences, General Surgery and Hepatobiliary Unit, Fondazione Policlinico Universitario Agostino Gemelli IRCCS, 00168 Rome, Italy; felice.giuliante@unicatt.it (F.G.); alfonsowolfango.avolio@unicatt.it (A.W.A.); 4 Department of Translational Medicine and Surgery, Università Cattolica del Sacro Cuore, 00168 Rome, Italy; 5 Department of Gastroenterological, Endocrine, Metabolic and Nephro-Urological Sciences, General Surgery and Transplantation Unit, Fondazione Policlinico Universitario Agostino Gemelli IRCCS, 00168 Rome, Italy

**Keywords:** liver transplant, hemoadsorption, graft failure, liver resection, liver dysfunction

## Abstract

**Background**: Hepatic dysfunction (HD) after liver transplantation (LT) or extended hepatic resection (EHR) is associated with graft failure and high short-term mortality. We evaluated the safety and depurative efficacy of CytoSorb® in these settings. The primary endpoint was the change in serum total bilirubin at the end of the treatment compared to the baseline value. The secondary endpoint was to evaluate the trend of serum total bilirubin and coagulation parameters up to 72 h after discontinuation of CytoSorb®. The effects of CytoSorb® therapy on the degree of hepatic encephalopathy (HE), Sequential Organ Failure Assessment (SOFA), and Model for End-Stage Liver Disease (MELD) scores as well as the hemodynamic status compared to baseline were also assessed. **Methods**: Adult patients with a serum total bilirubin level > 10 mg/dL admitted to the Intensive Care Unit were included. Exclusion criteria were hemodynamic instability, postoperative bleeding and platelet count < 20,000/mm3. **Results**: Seven patients were treated. Serum total bilirubin was significantly reduced at the end of treatment. However, seventy-two hours after the discontinuation of extracorporeal therapy, bilirubin levels returned to baseline levels in four patients. A decrease in platelet count was found during therapy, and platelet transfusion was required in six cases. A significant increase in D-dimer at the end of treatment was detected. HE degree, SOFA and MELD scores remained stable, while a deterioration in hemodynamic status was observed in two cases. **Conclusions**: Our preliminary findings did not show the possible benefits of CytoSorb^®^ in rebalancing clinical and laboratory parameters in patients with HD after LT or EHR.

## 1. Introduction

Hepatic dysfunction (HD) after liver transplantation (LT) or extended hepatic resection (EHR) is associated with graft failure and high short-term mortality [1,2]. Impaired hepatic detoxification, protein synthesis and metabolism lead to the accumulation of water-soluble, albumin-bound toxins and inflammatory mediators, which play a crucial role in the development of cardiovascular and neurotoxic complications, even leading to multi-organ failure (MOF) [3]. Several extracorporeal liver support systems have been tested in patients with HD as a bridge to recovery or LT [4,5]. The most widely used device is the molecular adsorbent recirculating system (MARS), which is designed to remove albumin-bound toxins. Despite its cleansing efficacy, a multicenter randomized controlled trial failed to demonstrate a beneficial effect on mid-term survival [6]. In addition, MARS was not effective in removing cytokines involved in the pathogenesis of MOF [3]. CytoSorb**^®^** is an innovative extracorporeal purification system (CytoSorbents Corporation, Princeton, NJ, USA) consisting of a cartridge containing biocompatible polystyrene-divinylbenzene copolymer beads. It removes from blood hydrophobic compounds with molecular weights up to 60 kDa. CytoSorb**^®^** is designed to remove cytokines and rebalance the inflammatory response in the MOF [7,8]. CytoSorb**^®^** hemoperfusion device has been used in patients with liver failure due to its ability to remove bilirubin and bile acids [9,10]. To date, there are limited data on the use of CytoSorb**^®^** in patients with HD following LT or EHR.

The primary endpoint was the change in serum total bilirubin at the end of the treatment compared to the baseline value (before starting CytoSorb**^®^** treatment). The secondary endpoint was to evaluate the trend of total bilirubin and coagulation parameters up to 72 h after discontinuation of CytoSorb**^®^**. Sustained improvement was defined as a stable or an unincreased bilirubin level during the 72 consecutive hours without extracorporeal therapy. We also assessed the effects of CytoSorb**^®^** therapy on the degree of hepatic encephalopathy (HE), Sequential Organ Failure Assessment (SOFA) and Model for End-Stage Liver Disease (MELD) scores, as well as hemodynamic status (reduction or increase in vasopressor support) compared to baseline.

## 2. Materials and Methods

Patients aged ≥ 18 years with a serum total bilirubin level higher than 10 mg/dL, admitted to the ICU of the Fondazione Policlinico Universitario A. Gemelli IRCSS, Rome, Italy, between January 2021 and May 2023 were retrospectively analyzed. This study was approved by the Institutional Ethics Committee (ID 6302). Written informed consent was obtained from each patient or their legal representative or next of kin. Inclusion criteria were total serum bilirubin levels above 10 mg/dL that remained persistently elevated or showed an increasing trend compared to pre-LT or pre-EHR levels despite maximal standard care and regardless of the presence of renal failure. Exclusion criteria were postoperative bleeding needing surgery, severe hemodynamic instability requiring high vasopressor support (norepinephrine > 1.0 µg/Kg/min), and platelet count < 20,000/mm^3^. HD after LT was defined according to early allograft failure simplified estimation (EASE) score and liver graft assessment following transplantation (L-GrAFT) score [11].

### 2.1. Extracorporeal Treatment Procedure

**Figure 1:**
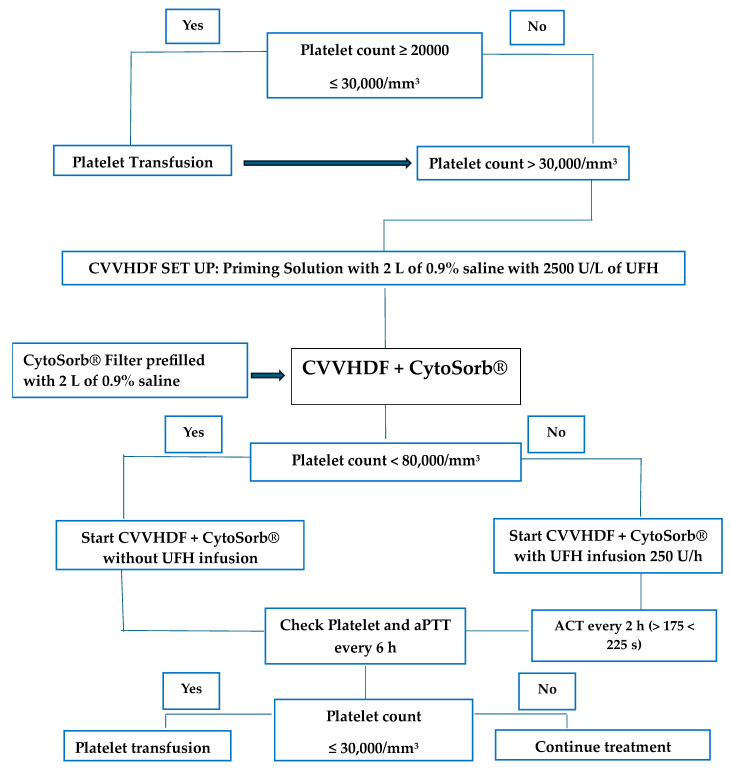
Management protocol during CytoSorb^®^ therapy. CVVHDF: continuous veno-venous hemodiafiltration; UFH: unfractionated heparin; ACT: activated clotting time.

The internal jugular vein or the femoral vein was cannulated with a 12 French high-flow triple lumen catheter (Arrowg+ard Blue-ARROW, Teleflex Medical Europe Ltd., Athlone Co Westmeath, Ireland). The CytoSorb**^®^** absorber was installed in-line in the continuous renal replacement therapy (CRRT) circuit (Prismaflex system, Baxter Medical AB, Kista, Sweden) at the post-hemofilter position). CRRT was performed in continuous veno-venous hemodiafiltration (CVVHDF) mode with a blood flow of 100–150 mL/min, substitution of 5–10 mL/Kg/h, dialysate 10–12 mL/Kg/h and ultrafiltration, as deemed appropriate by the prescribing physician to control fluid balance. A PrismaSol 2 solution (Baxter Medical AB, Kista, Sweden) was used as dialysate and replacement fluid. Two liters of 0.9% saline with 2500 U/L unfractionated heparin (UFH) was used as priming solution. Anticoagulation was performed using a continuous infusion of UFH in the CRRT circuit (at a starting dose of 250 U/h) to maintain an activated clotting time (ACT) between 175 and 225 s. The CytoSorb**^®^** filter was always pre-filled with two liters of 0.9% saline. Anticoagulation was not used if the platelet count was < 80,000/mm^3^. The risk of bleeding was mitigated with platelet transfusion before starting treatment to achieve a platelet count above 30,000/mm^3^. Only in these cases, our protocol included pretreatment of the Prismaflex filter (Baxter Healthcare Corporation, Chicago, IL, USA) with 2 L of heparinized priming solution. If no continuous anticoagulation was used, we tried to maintain the blood flow at the highest possible rate (Figure 1). CytoSorb**^®^** therapy was administered for a maximum of 24 h daily for three treatments over seven days. The entire course of three treatments was not completed if an organ was available for re-transplantation or if a severe hemodynamic instability requiring a significant increase in vasopressor (norepinephrine, >1.0 µg/Kg/min) or a critical reduction in platelet count occurred. Re-transplantation per se was considered a treatment failure.

### 2.2. Data Collection

Blood samples were collected at baseline (T0), at the end of treatment (T1) and 24, 48 and 72 h after discontinuation of CytoSorb**^®^** to measure selected blood biochemical and hematological parameters. SOFA [12] and MELD [13] scores and HE classified according to the West Haven criteria [14] were calculated at baseline and at the end of treatment. Demographic data collected at ICU admission included age, gender, body mass index, comorbidities (renal failure and/or respiratory failure requiring CRRT or mechanical ventilation) and liver disease diagnosis.

### 2.3. Statistical Analysis

Quantitative variables were described using the mean and confidence intervals. The Kolmogorov–Smirnov test was used to determine the normal distribution of data. We used the 2-side Student’s *t*-test to compare patients’ biochemical parameters’ values pre- and post-treatment. The “MEDcalc” version 18.6 was used for statistical analysis. A *p* value ≤ 0.05 was considered statistically significant.

## 3. Results

**Figure 2:**
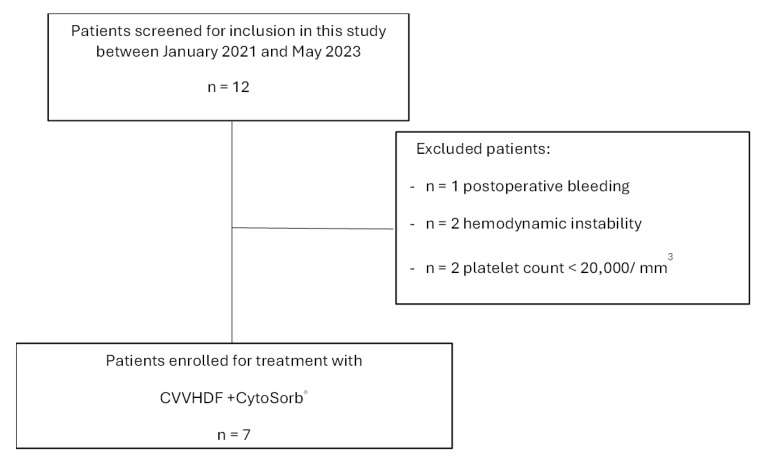
Flow diagram of the study.

The flow diagram of patients according to inclusion/exclusion criteria is shown in Figure 2.

Seven male patients with a median age of 60.4 (range: 49–75) years were treated. Five patients had graft dysfunction after deceased donor LT and two patients had post-hepatectomy liver failure after EHR. Before starting CytoSorb^®^, four patients required RRT, three patients received mechanical ventilation and one patient was treated with low doses of norepinephrine (<0.5 µg/Kg/min). Among the seven patients, two completed the cycle of three treatments. The remaining five patients received only two treatments for the following reasons: one patient underwent re-transplantation, two patients had hemodynamic instability requiring starting or increasing norepinephrine dose (>1.0 µg/Kg/min) and two patients developed severe thrombocytopenia despite multiple platelet transfusions. Demographic, clinical and biochemical variables at baseline and after treatment are shown in Table 1.

The changes in the assessed laboratory parameters at the end of the extracorporeal treatment compared to the baseline values are shown in Table 2.

**Figure 3:**
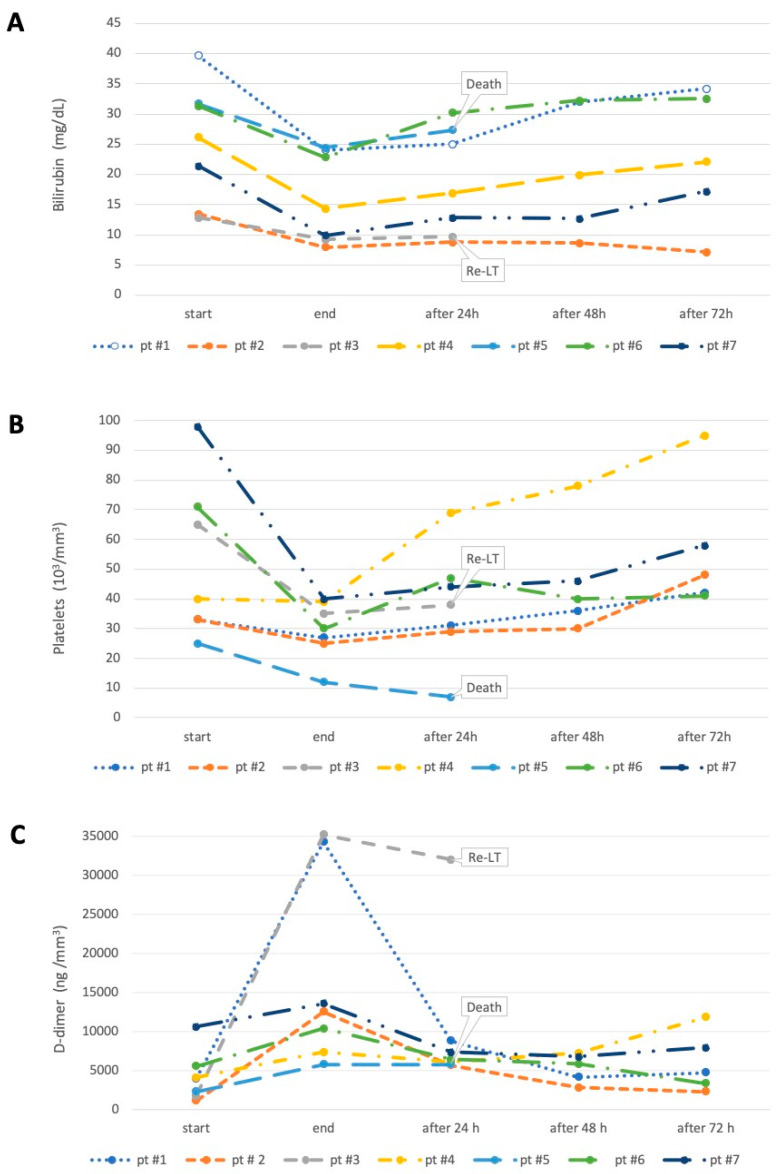
Comparison of biochemical parameters (A: bilirubin; B: Platelets; C: D-dimer) at the beginning, at the end of treatment, 24, 48 and 72 h, after CytoSorb^®^ treatment. re-LT: re-transplant; pt: patient.

Significant reductions in serum levels of bilirubin, alkaline phosphatase, urea nitrogen and creatinine were observed in all patients at the end of treatment. Plasma ammonia levels decreased in six patients and increased to 454 μg/dL in the patient scheduled for re-transplantation without neurological sequelae. We observed a significant decrease in platelet count in all patients from the first treatment, and six out of seven patients required platelet transfusions. All patients had a decrease in hemoglobin levels and required red blood cell transfusion during treatment, with no apparent blood loss. Finally, we detected a significant increase in international normalized ratio (INR), activated partial thromboplastin time and D-dimer in all patients. MELD and SOFA scores remained stable or changed slightly as an increased INR offset a decreased bilirubin level. The degree of HE remained unchanged in all cases. Total bilirubin, platelet count and D-dimer values at baseline, end of treatment and at 24, 48 and 72 h are shown in Figure 3. Notably, bilirubin levels decreased in all patients at the end of treatment. Bilirubin levels showed a return to the baseline levels in four patients and a modest reduction in only one patient 72 h after discontinuation of CytoSorb^®^. In two patients, the bilirubin trend was assessed only for the first 24 h as one patient was re-transplanted and another died (see Figure 3). In five patients, platelet counts and D-dimer levels returned to baseline 72 h after discontinuation of treatment. At 28 days, five patients were alive, including the re-transplanted patient, while two patients had died.

## 4. Discussion

Several new adsorbent devices have been developed to remove protein-related substances that accumulate dangerously in the blood of patients with impaired liver function. Hydrophobic substances such as conjugated bilirubin, bile acids, phenol, aromatic amino acids and fatty acids are not removed by conventional dialysis and can reach abnormally high blood levels with toxic effects on both the liver and other organs. Several adsorption devices have been proposed to remove these albumin-bound substances and water-soluble compounds such as ammonium. The new absorbent system, CytoSorb^®^, was designed to remove cytokines and rebalance the inflammatory response in patients with MOF. The device has a suspended column of highly porous resin beads covered with a biocompatible coating. CytoSorb^®^ cartridges can be used in stand-alone mode (hemoperfusion) or inserted within a continuous renal replacement therapy circuit (associated with a conventional hemofilter), a cardiopulmonary bypass or an extracorporeal membrane oxygenation circuit. CytoSorb^®^ removes a wide range of compounds with molecular weights up to 60 kDa from the blood: pro- and anti-inflammatory cytokines, bilirubin, myoglobin, exotoxins and drugs. Its use has considerably expanded over recent years, with 211 centers currently participating in an international CytoSorb^®^ registry.

Cytokine adsorption using CytoSorb^®^ has been proposed in various clinical settings, including sepsis, acute respiratory distress syndrome, hyperinflammatory syndromes, cardiac surgery or recovery after cardiac arrest. In a recent review, the authors conclude that there is currently no high-quality evidence to justify the widespread use of the CytoSorb^®^ adsorber in critical care medicine [15,16].

In the hepatological field, including acute or acute on chronic liver failure, liver transplantation and liver resection surgery, the number of studies is still very limited.

Similar to other authors [9,10,17,18,19], we observed a reduction in serum bilirubin levels after CytoSorb^®^ treatment. However, in our case series, this reduction was transient. To date, only one study has reported a trend of bilirubin levels after discontinuation of CytoSorb^®^ and, in particular, up to 72 h after its withdrawal [20]. We observed a return of bilirubin levels to the baseline levels in four patients and a small reduction in only one patient. In two patients, bilirubin trends were not assessed until 72 h due to re-transplantation or death within the first 24 h after discontinuation. In our patients, the recovery of baseline bilirubin levels appeared to be consistent with a lack of improvement in liver function. We also observed a significant decrease in the platelet count, as reported by other authors, in patients who underwent hemoadsorption for “hepatic indications” [9,10,18]. In our patients, the decrease in platelet count was clinically substantial and required multiple transfusions, with a potential negative impact exerted on clinical outcomes, including an increased occurrence of severe complications and higher mortality [21,22,23,24,25,26,27]. Thrombocytopenia was also the most frequent adverse event in a large cohort of patients treated with MARS^®^ therapy [28]. However, in our case series, the thrombocytopenia was possibly not due to the treatment of CytoSorb^®^ alone, but to its combination with renal replacement therapy [29,30,31,32,33]. The decrease in platelet count may also be caused by the heparin used for anticoagulation of the extracorporeal circuit. However, in our series, only one patient received heparin for the entire duration of treatment. In the remaining cases, heparin was used only in the priming solution, or the continuous infusion was discontinued during treatment due to a marked decrease in platelet count. Further studies comparing CRRT alone with CRRT plus CytoSorb^®^ are needed to determine whether the reduction in platelet count is more relevant with the addition of CytoSorb^®^.

Given the extent of the platelet reduction and the increase in INR and aPTT, CytoSorb^®^ treatment needs to be carefully evaluated in patients with liver failure, who are often severely thrombocytopenic and do not require hemodialysis.

As recently reported, there is currently a lack of data on D-dimer assessment before and after hemoadsorption treatment [19]. In contrast to previous studies [9,10,17,18,19], we first reported an increase in D-dimer levels during CytoSorb^®^ treatment, which tended to return to baseline values 72 h after discontinuation. It is well known that D-dimers are mini-fibrin protein fragments. They result from the natural degradation of clots (composed of fibrin and platelet networks) by plasmin in the body. Therefore, the presence of fibrinolysis is indicated by the detection of circulating D-dimers. The contact of blood with extracorporeal surfaces has a thrombogenic effect [34,35]. In our patients, the dissolution of clots formed during hemodialysis may have caused the increase in D-dimer detected at the end of extracorporeal treatment. Moreover, the decrease in fibrinogen level at the end of treatment may indicate the activation of the coagulation cascade. In routine clinical practice, unfractionated heparin is used to prevent clot formation in the extracorporeal circuit. In patients with liver failure, the therapeutic window of anticoagulants is limited. Excessive inhibition of the coagulation cascade may expose the patient to the risk of bleeding. We used heparin infusion at low doses during treatment only in patients with platelet count ≥ 80,000/mm^3^. We do not have sufficient data to determine whether the absence or reduced use of anticoagulant in the extracorporeal circuit caused an increase in clot formation and consequently an increase in D-dimer levels, or whether this increase was due to CytoSorb^®^ therapy or to the extracorporeal treatment itself, regardless of the use of anticoagulants. Increased D-dimer levels may be the focus of further studies.

In our case series, ammonia levels did not change significantly, and the degree of hepatic encephalopathy remained stable in all patients. In the patient scheduled for re-transplantation, we observed an increase in ammonia levels during treatment, but this did not lead to neurological sequelae. Notably, in our experience, CytoSorb^®^ treatment did not reduce scores indicating organ dysfunction because the relevant reduction in bilirubin and creatinine was “compensated” by the severe decrease in platelet count for the SOFA score and the increase in INR for the MELD scores.

### Strengths and Limitations

This study included a poorly explored population of patients with liver hepatic dysfunction, admitted to the ICU after liver transplantation or extensive hepatic resection. Nevertheless, this study has important limitations. First, the small number of cases; second, the availability of inflammatory cytokine measurements in only two patients; and third, the lack of a control group.

## 5. Conclusions

We suggest caution in using CytoSorb^®^ in thrombocytopenic patients with liver failure because of its possible detrimental effects on hemostasis. In addition, it remains to be determined whether non-selective blood purification may be beneficial for the removal of ‘bad toxins’, or whether it may be detrimental for the alteration of coagulation physiology. Pending the publication of findings derived from the CytoSorb^®^ registry, the use of this device in patients post liver surgery/transplantation needs to proceed with caution, especially in the case of thrombocytopenia.

## Figures and Tables

**Table 1:** Demographic, clinical and biochemical parameters of patients before and after CytoSorb^®^ treatment.

Variable	Case 1	Case 2	Case 3	Case 4	Case 5	Case 6	Case 7
Age, years	58	49	56	53	63	75	69
Sex, male/female	Male	Male	Male	Male	Male	Male	Male
Body mass index, Kg/m^2^	26.9	23.2	21.9	22.5	21.5	27.7	21.5
Etiology of liver disease	GD	GD	Severe GD	GD	GD	PHLF	PHLF
CytoSorb^®^ session, N	3	2	2	2	2	2	3
RRT	No	Yes	Yes	Yes	Yes	No	No
Mechanical ventilation	No	No	Yes	Yes	Yes	No	No
Time from surgery, days	7	5	2	10	26	17	23
° Parameter at T0/T1	Case 1	Case 2	Case 3	Case 4	Case 5	Case 6	Case 7
HE grade	II/II	I/I	IV/IV	II/II	II/II	I/I	I/I
SOFA score	9/10	8/7	18/17	10/10	14/14	7/9	6/6
MELD score	25/24	32/31	40/40	35/32	37/37	24/24	18/18
Vasopressor therapy	no/no	no/no	yes/yes	no/no	yes/yes ^+^	no/yes	no/no
Total bilirubin, mg/mL	39.7/24.0	13.4/7.9	12.8/9.2	26.1/14.3	31.7/24.4	31.3/22.8	21.4/9.9
Creatinine, mg/mL	0.7/0.5	1.7/1.2	2.2/1.6	1.8/0.8	0.6/0.4	1.1/1.0	0.6/0.5
Ammonia, µg/dL	79/36	142/74	398/454	45/32	67/29	93/90	52/47
Bile acid, μg/mL	-	41.2/8.5	-	-	-	97/93	-
Arterial lactate, mmol/L	1.0/1.0	0.6/1.2	5.0/2.9	1.4/1.4	0.9/1.1	1.8/2.0	0.9/0.9
AST, U/L	40/25	29/37	1117/352	50/47	40/47	185/202	192/160
ALT, U/L	42/17	168/90	2296/793	52/13	13/13	107/103	238/150
ALP, U/L	94/78	163/93	97/87	179/148	500/435	110/112	286/224
GGT, U/L	117/66	183/53	59/41	158/127	71/64	108/100	161/135
Hemoglobin, g/dL	9.2/8.0 ^	9.1/9.0	9.3/8.2	8.4/8.1	11.0/11.2	9.5/9.1	8.5/8.3 ^
Platelets count, ×10^3^/mm^3^	33/27 *	33/25 *	65/35 *	40/39 *	25/12 *	71/30 *	98/65
Leucocytes, ×10^3^/mm^3^	12.7/19.5	2.3/3.4	9.3/11.8	5.8/6.2	6.2/3.5	15.0/9.3	10.3/5.9
INR	1.5/1.7	1.3/1.4	3.1/3.5	1.2/1.3	1.5/1.6	1.4/1.6	1.0/1.3
aPTT, s	40.7/83.7	33.7/52.2	60.2/91.0	48.0/49.2	70.5/76.6	67.0/93.0	47.9/58.2
D-dimer, ng/mm^3^	3926/ 34,308	1076/ 12,508	1760/ 35,200	4084/ 7336	2263/ 5732	5523/ 10,354	10,605/ 13,572
Fibrinogen, mg/mm^3^	201/180	190/195	179/166	379/326	403/354	261/226	597/518
Interleukine-6, pg/mL	-	-	-	-	-	15.9/29.3	94.2/59.1
28 days outcome	Alive	Alive	Alive re-LT	Alive	Dead	Dead	Alive

**°**: T0/T1: values before starting (T0) and after ending (T1) of CytoSorb^®^ therapy; ^+^: increased dose; ^: red blood cell transfusion; *: platelet transfusion. Abbreviations: GD, graft dysfunction; PHLF: post-hepatectomy liver failure; RRT: renal replacement therapy; HE, hepatic encephalopathy; SOFA: Sequential Organ Failure Assessment; MELD: Model for End-Stage Liver Disease; AST: aspartate aminotransferase ALT, alanine aminotransferase; ALP, alkaline phosphatase; GGT, gamma-glutamyl transferase; INR: international normalized ratio; aPTT: activated partial thromboplastin time; re-LT: re-transplant.

**Table 2:** Comparison between laboratory parameters before and after CytoSorb^®^ treatment. Values are means (95% confidence intervals). Bold values denote statistical significance at the p < 0.05 level.

Variable	Pre-Treatment	Post-Treatment	*p*
Creatinine, mg/mL	1.24 (0.6–1.8)	0.86 (0.5–1.2)	**0.006**
Urea nitrogen, mg/dL	45 (22.6–66.1)	30 (14.3–45.3)	**0.01**
Arterial lactate, mmol/L	1.6 (0.6–2.3)	1.37 (0.7–2.2)	0.89
AST, U/L	233.4 (26.1–325.9)	133.14 (12.3–253.9)	0.52
ALT, U/L	404.0 (24.4–480.5)	175 (21.3–273.7)	0.11
Total bilirubin, mg/mL	25.2 (15.5–34.9)	16.1 (9.1–22.9)	**0.001**
Conjugated bilirubin, mg/mL	18.9 (11.5–26.5)	12.3 (7.3–17.2)	**0.005**
GGT, U/L	118.7 (75.8–162.2)	87.43 (46.9–127.8)	0.10
ALP, U/L	204.1 (96.2–302.0)	168.2 (49.7–286.4)	**0.02**
Ammonia, µg/dL	123.4 (45.3–184.5)	122.17 (26.5–205.8)	0.10
Hemoglobin, g/dL	9.3 (8.5–10.0)	9.5 (8.3–10.6)	0.68
Platelets count, ×10^3^/mm^3^	49.4 (28.6–69.2)	30.86 (20.8–40.8)	**0.02**
Leucocytes, ×10^3^/mm^3^	8.8 (4.2–13.6)	8.5 (3.2–13.8)	0.70
INR	1.6 (1.0–2.0)	1.8 (1.0–2.4)	**0.02**
aPTT, s	52.57 (39.7–65.4)	73.94 (54.1–93.7)	**0.01**
Fibrinogen, mg/mm^3^	315.7 (188.3–439.2)	286.86 (174.1–399.5)	0.16
D-dimer, ng/mm^3^	4176 (1615–6631)	17001 (5504–28,498)	**0.01**

Abbreviations: AST, aspartate aminotransferase ALT, alanine aminotransferase; GGT, gamma-glutamyl transferase; ALP, alkaline phosphatase; INR: international normalized ratio; aPTT: activated partial thromboplastin time.

## Data Availability

The data that support the findings of this study are available from RG, upon reasonable request.
